# Physiological and Multi-Omics Approaches for Explaining Drought Stress Tolerance and Supporting Sustainable Production of Rice

**DOI:** 10.3389/fpls.2021.803603

**Published:** 2022-01-27

**Authors:** Sajad Majeed Zargar, Rakeeb Ahmad Mir, Leonard Barnabas Ebinezer, Antonio Masi, Ammarah Hami, Madhiya Manzoor, Romesh K. Salgotra, Najeebul Rehman Sofi, Roohi Mushtaq, Jai Singh Rohila, Randeep Rakwal

**Affiliations:** ^1^Proteomics Laboratory, Division of Plant Biotechnology, Sher-e-Kashmir University of Agricultural Sciences and Technology of Kashmir, Srinagar, India; ^2^Department of Biotechnology, School of Biosciences and Biotechnology, BGSB University, Rajouri, India; ^3^Department of Agronomy, Food, Natural Resources, Animals, and Environment, University of Padova, Padua, Italy; ^4^School of Biotechnology, Sher-e-Kashmir University of Agricultural Sciences and Technology of Jammu, Jammu, India; ^5^Division of Plant Breeding and Genetics, Sher-e-Kashmir University of Agricultural Sciences and Technology of Kashmir, Srinagar, India; ^6^Department of Biotechnology and Bioinformatics, SP College, Cluster University Srinagar, Srinagar, India; ^7^Dale Bumpers National Rice Research Center, United States Department of Agriculture (USDA)-Agricultural Research Service (ARS), Stuttgart, AR, United States; ^8^Faculty of Health and Sport Sciences, University of Tsukuba, Ibaraki, Japan

**Keywords:** QTL, global food security, multiomics, rice, drought, abiotic stress, proteome, metabolome

## Abstract

Drought differs from other natural disasters in several respects, largely because of the complexity of a crop’s response to it and also because we have the least understanding of a crop’s inductive mechanism for addressing drought tolerance among all abiotic stressors. Overall, the growth and productivity of crops at a global level is now thought to be an issue that is more severe and arises more frequently due to climatic change-induced drought stress. Among the major crops, rice is a frontline staple cereal crop of the developing world and is critical to sustaining populations on a daily basis. Worldwide, studies have reported a reduction in rice productivity over the years as a consequence of drought. Plants are evolutionarily primed to withstand a substantial number of environmental cues by undergoing a wide range of changes at the molecular level, involving gene, protein and metabolite interactions to protect the growing plant. Currently, an in-depth, precise and systemic understanding of fundamental biological and cellular mechanisms activated by crop plants during stress is accomplished by an umbrella of -omics technologies, such as transcriptomics, metabolomics and proteomics. This combination of multi-omics approaches provides a comprehensive understanding of cellular dynamics during drought or other stress conditions in comparison to a single -omics approach. Thus a greater need to utilize information (big-omics data) from various molecular pathways to develop drought-resilient crop varieties for cultivation in ever-changing climatic conditions. This review article is focused on assembling current peer-reviewed published knowledge on the use of multi-omics approaches toward expediting the development of drought-tolerant rice plants for sustainable rice production and realizing global food security.

## Introduction

Rice (*Oryza sativa* L.) is the topmost grain consumed as a staple food by humans worldwide, most prominently in Asian countries (Asia, 2021; Samal et al., 2021). It belongs to the family *Poaceae* and tribe *Oryzeae* and presently comprises 22 wild species with only two species cultivated at a global level. Rice, with a genome size of 430 Mb, is an important model crop plant, and it has been reported that Asia ranks the highest in global rice production (Manavalan et al., 2012; Asia, 2021). As rice requires more water, up to 4000 liters per kilogram production, the plant is more prone to drought stress than other environmental stressors. Among all these stressors, drought stress is a major factor that can hinders the growth, yield and productivity of rice crops (Mumtaz et al., 2020). Drought is a multi-faceted stress and may affect rice production in various ways. In rainfed areas, it affects directly by further drying of the paddy soils, but in irrigated areas it hits in the form of declining underground water tables and become an issue for water sustainability. In the field, drought effects can be compounded by interactions with other biotic and abiotic stresses. Further, conventional cultural practices in rice require a season-long flooding, but in today’s circumstances, it is unsustainable, and there is competition for the utilization of surface water (rivers, etc.) with increased urbanization; additionally, low underground water availability due to the continually declining water tables of aquifers is also becoming common. All these combinations have increased a strong need for understanding and increasing drought tolerance in rice crop.

The negative implications of drought stress on the physiological functioning of plants are mainly due to its reduced water potential and turgor pressure that suppress plant growth and metabolism (Lisar et al., 2012). Drought is a period in crop’s growing season during which the soil moisture reaches to a certain level that reduces its yield or quality resulting from either limited irrigations or a below average rate of precipitation and higher evapo-transpirations, which causes a decline in plant growth and productivity (Rollins et al., 2013; McClung et al., 2020). Drought affects plants in numerous ways by impairing normal molecular, metabolic and physiological networks to reduce growth and metabolism (Zu et al., 2017). A typical implication of drought includes a decline in the expansion of leaves and an overall decrease in the stomatal conductance and rate of photosynthesis (Avramova et al., 2016; Barnaby et al., 2019). Furthermore, a reduction in growth is also reported to be induced by the inhibition of cell elongation, cell expansion and impairment in mitosis (Potopova et al., 2016). Against these consequences, plants respond at the morphological, physiological and molecular levels. These responses include maximum uptake of water by a dense and deep root system, reduction in water loss by closure of stomata, adjustments in osmosis, reducing the leaf area, altering the elasticity of cell wall and several more physiological adjustments (Saha et al., 2016). Physiological traits are critical to selecting drought-tolerant germplasm of crop plants, even though the response to drought stress by crop plants is dependent on the environment and diverges across genotypes and genetic interactions (Lonbani and Arzani, 2011).

At present, freshwater shortage is major issue on global scale and could be more critical in the near future as per climate fluctuation projections. In developing sustainable solutions to overcome water scarcity, there is a constant need for scouting strategies that could be possible alternatives to ensure the availability and accessibility of freshwater to crop plants (Noemi et al., 2014). Rice, among other cereal crops, demands access to freshwater, i.e., water-intensive cultivation. Moreover, half of humankind relies on rice as a staple food, and at the global level, a total of 160 million hectares (Mha) of cultivated land comprises this crop, most of which (around 40%) lies in Asian countries (Prasad et al., 2017). The rice plant is cultivated in flooded paddy fields since it requires an adequate water supply of almost 2 to 3 times more than dryland cereals.

Consequently, researchers are now faced with a challenge to increase the adaptation of rice cultivars to water-scarce conditions and to reduce the dependency on a large quantity of water to support their cultivation in almost dry land cultivation systems (Sandhu et al., 2014). Major adaptations include adjustments in physiological, anatomical and morphological characteristics of root and shoot traits (Kadam et al., 2015; Sandhu et al., 2016) (Tables 1,2). An irrigated rice system is the most commonly cultivated practice, comprising up to 55% of the 158 Mha cultivated land area, whereas 34% of the 54 Mha land cover is shared by rainfed lowland systems, and rice grown in flood-prone areas shares 7% of the 11 Mha land area (Bouman et al., 2007). Approximately 37% of global rice production is from South Asia, among which 50% is rainfed (Dawe et al., 2010). Moreover, rainfed rice is also cultivated in sub-Saharan Africa, accounting for up to 84% of the total area of rice cultivation (Gauchan and Pandey, 2012). Since the global production of rice is grossly dependent on rainfed ecosystems, drought stress becomes a major factor behind decreased productivity from 13 to 35%. Thus, one can witness an enormous global loss in rice productivity compared to other crops due to its higher dependency on water and hence proneness to drought stress. All the evidence suggests that climate induced fluctuating rainfall and drought result in heavy losses to rice farming systems (Fahad et al., 2019).

**TABLE 1:** Drought tolerance genes that have been tested on rice.

Sr. No	Cellular mechanism	Gene	Promoter	Genetic transformation method	Targeted phenotype	References
1	Abscisic acid metabolism	CAMV35SP	DSM2	Agrobacterium	Oxidative and drought stress tolerance	Du et al., 2010
2	ROS scavenging	OSSROIC	Ubi 1	Agrobacterium	Oxidative stress tolerance and stomata closure regulation	You et al., 2013
3	Protoporphyrinogen oxidase	PPO	−	Agrobacterium	Less oxidative damage and drought tolerance	Phung et al., 2011
4	Ubiquitin ligase	OSSDIR1	CAMV35 Ubi1	Agrobacterium	Stomata regulation under drought stress	Gao et al., 2011
5	Abscisic acid sensitivity	OSSAPK2	−	−	Abscisic acid sensitivity and drought tolerance	Lou et al., 2017
6	DNA damage repair and defense response	OsNAC14	OsRAD51A1		DNA damage repair and defense response resulting in improved tolerance to drought	Shim et al., 2018
**7**	Multiple stress tolerances in rice plants during both seedling and panicle development stages	OsAHL1	P*_*AHL1*_*	−	Regulates root development under drought condition to enhance drought avoidance	Zhou et al., 2016
8	Enhanced resistance to a bacterial pathogen	*Os*WRKY11	CHIT 2	−	Enhanced resistance to a bacterial pathogen	Lee et al., 2018
9	Reactive oxygen species scavenging	OsLG3	*OsLG3-OE*	−	Reactive oxygen species scavenging and drought tolerance	Xiong et al., 2018
10	Increased moderate susceptibility to the pathogens	*OsMADS26*	ubiquitin1	−	Increased moderate susceptibility to the pathogens and drought tolerance	Khong et al., 2015
11	Induced a variety of environmental stresses and plant hormones	OsDRAP1	CaMV35S	Agrobacterium	High expression in response to drought	Huang et al., 2018
12	Increases ABA sensitivity and enhances osmotic tolerance in rice	*OsEm1*	LEA	−	ABA sensitivity and enhances osmotic tolerance promising for engineering drought tolerance in rice	Yu et al., 2016
13	Reactive oxygen species (ROS)-scavenging	OsCML4	CaMV35S	−	Reactive oxygen species (ROS)-scavenging and drought tolerance	Yin et al., 2015
14	Control of tiller outgrowth	*OsIAA6*	PGD1	Agrobacterium	Drought stress responses and the control of tiller outgrowth.	Jung et al., 2015
15	Conversion of aspartate amino acid to glutamate was found to be associated with drought tolerance	*OsDREB1F*	CaMV35S		The categorization of all the significant SNPs with H5 drought tolerant haplogroup supports their role in drought tolerance in rice	Singh et al., 2015

**TABLE 2:** QTLs identified for drought tolerance related traits in rice.

S. No	Targeted trait	Number of QTL’S	References
1	Grain yield	1 (Qdty2.1)	Mishra et al., 2013
2	Grain yield	1 (Qdty3.2)	Yadaw et al., 2013
3	Grain yield	14	Wang et al., 2014
4	Filled grain number per panicle	23	Wang et al., 2014
5	Panicle number per plant	14	Wang et al., 2014
6	Grain yield	1 (Qdty2.3)	Palanog et al., 2014
7	Grain yield	1 (Qdty2.2)	Palanog et al., 2014
8	Grain yield	4	Saikumar et al., 2014
9	Grain yield	7	Singh et al., 2015
10	Grain yield	1 (qDTY 12.1)	Swamy et al., 2018
11	Flowering time	1	Prince et al., 2015
12	Flowering time	5	Saikumar et al., 2014
13	Flowering time	1	Sandhu et al., 2014
14	Canopy temperature	6	Prince et al., 2015
15	Biomass	8	Prince et al., 2015
16	Biomass	4	Saikumar et al., 2014
17	Drought index	3	Prince et al., 2015
18	Grain weight	2	Zhou et al., 2013
19	Grain yield	24	Verma et al., 2014
20	Seed setting rate	6	Prince et al., 2015

A sustainable approach for rice production is to minimize irrigation, which is being realized in commercial production by the introduction of alternate wetting and drying (AWD) irrigation management (de Avila et al., 2015; Chen M.-H. et al., 2021; see also^1^). Currently, farmers are practicing safe-AWD levels because most current varieties are bred for season-long flood irrigation management under both lowland and upland rice cultivations. In AWD, there is a drying phase, and rice is susceptible to drying conditions, because they affect yield and grain quality (McClung et al., 2020). Shaibu et al. (2015) compared Nunkile and NERICA 4 rice varieties, which are adapted to upland and lowland irrigated conditions, under continuous flood and three different AWD schemes. The results showed that water productivity for continuous flood treatment was superior to all three AWD schemes. Hence, for significant water savings, farmers need improved varieties that can withstand drier soils without decreasing yield and grain quality. Thus, to develop improved rice cultivars that can produce profitable yields with significant water savings, researchers need a better understanding of drought tolerance mechanisms and of the tools available to them. Furthermore, the situation is compounded by the use of susceptible rice varieties by farmers in subtropical and tropical cultivated areas (Zu et al., 2017). Thus, it is important to develop new rice cultivars that possess traits such as higher yield and resilience to drought stress. The evolution and production of drought tolerant rice varieties are developed by understanding the molecular mechanisms and signal responses initiated by tolerant cultivars under water deficit conditions. This approach does not mean that the traditional germplasm or breeding line selection process does not play an important role in this process of developing new rice varieties, some of which could have desirable traits such as short duration with higher yield (Mehana et al., 2021). However, the underlying molecular mechanism must be understood at a holistic level, and therein omics tools are needed and are prominent.

**FIGURE 1:**
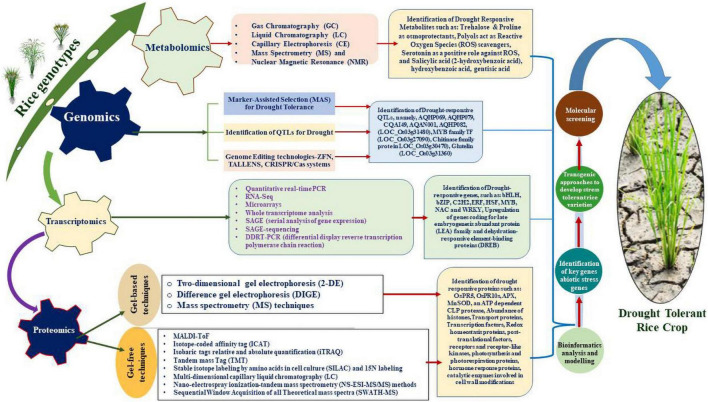
Diagrammatic representation of OMICS approaches employed to unravel the genes/proteins to produce transgenic rice plants.

The past few decades have witnessed extensive research based on application of omics technologies paving way to identify a large number of candidate genes, proteins and pathways to generate drought tolerant varieties of rice plants (Panda et al., 2021; Figure 1). Omics-based high throughput techniques facilitated unbiased studies on the genome, epigenome (epi)transcriptome, metabolome and proteome (Oliver et al., 1998; Hasin et al., 2017; Yu et al., 2021). Furthermore, technologies are employed to comprehend the underlying molecular mechanisms to resolve complex cellular responses and effects on the phenotypes of the crop (Manzoni et al., 2018; Upadhyaya and Panda, 2019). Considerable molecular data has been created by advancements in genomic and transcriptomic techniques but researchers are still behind in correlating the data with the proteomes due to limited depth of quantitative proteomic data inputs with respect to post-translational modifications especially (Molina et al., 2011; Ghatak et al., 2017). To date, gene expression analyses through applications of high-throughput technologies have revealed the transcription levels in cells. In addition, several investigations were conducted to correlate the transcriptomic and proteomic information to highlight the critical impact of post-transcriptional modifications on cellular subtleties (Foss et al., 2011; Ghazalpour et al., 2011). Recent advancements in drought stress-related data support the role of omics technologies in understanding and determining the mechanistic make-up of plants, to develop crop varieties that are resilient to drought stress. Most of these studies have demonstrated the critical role of post-transcriptional and post-translational modifications of proteins in defensive mechanisms that allow the plant to adapt to a diverse range of abiotic stressors (Matsuura et al., 2010; Sormani et al., 2011; Juntawong and Bailey-Serres, 2012; Liu et al., 2012).

The quantification of biomolecules, such as genes and proteins, is now easier due to the advent of high-throughput technologies, for studying the transcriptomes and proteomes. For instance, the E3-Ubiquitin Ligases proteins abundantly expressed in rice help in modulation of abiotic stressors such as drought, salinity, nutrient deprivation and radiation (Melo et al., 2021). Drought tolerance genes and their cellular processes, such as signaling-controlled gene expression and cellular modifications, have been deeply investigated by multi-omics high-throughput technologies (Huynh et al., 2018; Yadav et al., 2019; Singh et al., 2021). Keeping in perspective the importance of a multi-omics approach to better understand abiotic stress tolerance mechanisms in crops and developing novel stress mitigation strategies, the present article comprehensively summarizes the state-of -the-art knowledge on the molecular aspects of drought stress. Moreover, the current review also presents insights gleaned thus far on drought stress using various omics approaches with a special focus on rice, given its global significance as a major food crop.

## Approaches in Use for Conferring Drought Tolerance in Rice

The improvement of rice varieties could be accomplished by mining genes and superior alleles capable of better signal perception, signal transduction, and functional roles in drought tolerance. The repository of resilient genes and traditional donors of these genes includes wild accessions, landraces and varieties, such as Aus 276, Birsa gora, Dhagaddeshi, Dular, Kali Aus, Nagina 22 (or N22), and Vandana. Considering genetic improvement, several varieties have been developed through the selection of resilient landraces for a specific trait. For example, the landrace “Rajbhog” found in the foothills of Nepal was used to develop Nagina 22 through selection processes and is also a well-known drought tolerant variety of rice (Vikram et al., 2011). Furthermore, a drought tolerant Kataush landrace from the Nepal Tarai region was characterized (Puri et al., 2010). Landraces also resulted in the development of Laloo-14, a drought tolerant Indian rice variety cultivated in rainfed areas of Madhya Pradesh state of India, which validates the immense potential of landraces for improving rice cultivars for cultivation in rainfed areas (Akshaya et al., 2017). The rich repository of resilient genes found in landraces makes them suitable candidates to look for adaptation related genes or superior alleles important for resiliency for production in limited irrigation management. Various drought tolerant Indian landraces have been reported to possess drought tolerant genes (Vanniarajan et al., 2012). For further improvement of rice crop, it is pertinent to also identify and characterize drought tolerant genes by modern molecular breeding technologies.

**FIGURE 2:**
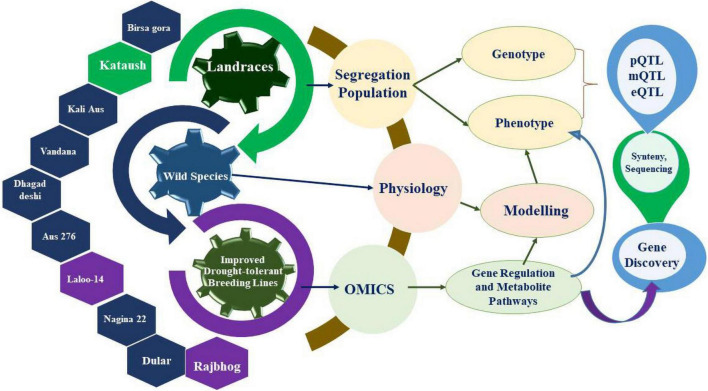
Illustration of drought tolerant gene discovery in parental lines of rice plants by combined approaches of omics and physiological methods.

Apart from the landrace varieties and wild germplasm accessions, natural hybrids and genetic stocks also act as rich genetic sources for the improvement of rice cultivars (Biswas et al., 2020). This wide range of rice landraces can be used to identify genes by diverse approaches, including omics-based techniques (Figure 2), for their subsequent transfer into elite breeding lines.

Only a few traits that pertain to roles in water use efficiency (WUE) and tolerance to drought have been implemented in the field, even though numerous crop species have been screened by mining drought tolerant genes (Langridge and Reynolds, 2015). A wide range of techniques, such as omics tools, molecular breeding and precise phenotyping, augment the identification of candidate genes that pertain to metabolic and signaling pathways and that are pivotal for drought tolerance in crops. Besides, the workflows for initiating the in-depth cellular phenotyping of diverse crop plants through applications of multi-omics technologies will further augment the quest of unraveling the resilience mechanisms (Zivy et al., 2015). In addition, various studies, such as functional genomics and expression profiling of genes, are available to comprehend the complexity of drought tolerance mechanisms in crop plants (Delphine et al., 2010; Li et al., 2018). Differential gene expression analysis has also been used to identify drought responsive genes in crop plants (Guo et al., 2008; Gray et al., 2010). Even though realistic progress through omics technologies such as metabolomics, proteomics, and genomic methods compared with epigenomics methodologies is scant, these approaches have provided much needed and useful information pertaining to genetic and physiological attributes linked to drought tolerance. We aim to combine efforts in the application of omics approaches in conjunction with plant breeding methods to extend the development of resilient rice cultivars aimed at cultivation in rainfed ecological niches or save significant amounts of water under irrigated field managements.

### Plant Traits

**FIGURE 3:**
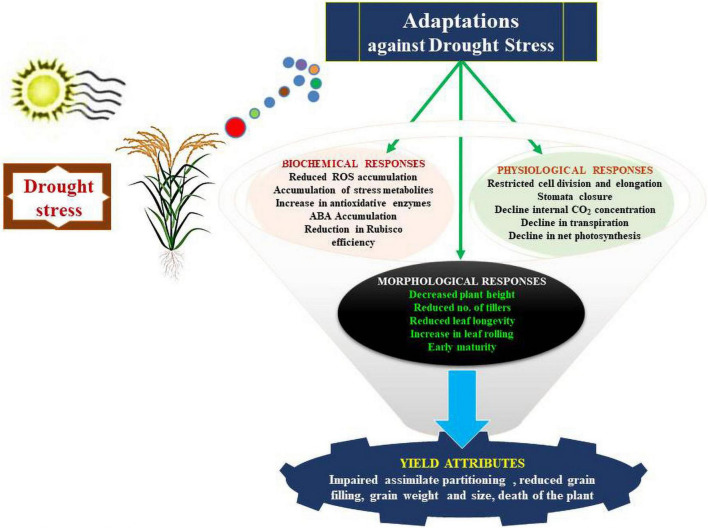
Series of physiological responses that allow plants to adapt to environmental conditions that induce drought stress.

Physiological adjustments established by crop plants against drought stress enable them to regulate their water requirements for normal physiology, growth and metabolism (Figure 3). The following sub-sections detail the important physiological adjustments accomplished by crop plants against drought stress:

### Shoot Traits

Reductions in growth and metabolism are primarily induced by drought in rice plants. Moreover, the initiation of new aerial organs, such as the stems, leaves and pre-existing organs, in aerial parts of plants are considerably affected by drought stress (Chaves et al., 2002). Central to the reduced growth of these aerial parts is the decline in cell division in plants and modulation of the physio-chemical properties of cell walls due to an increase in cell wall rigidity, to reduce expansion and hence the growth (Granier et al., 2000; Cosgrove, 2005). A significant decline in the leaf expansion rate was observed when water stress of 80% transpirable water soil moisture for 20 days was observed in the vegetative phase in rice (Farooq et al., 2009). Thus, adaptations result in limited loss of water by transpiration through leaf surfaces. A reduction in the rate of photosynthesis in rice prevails based on the change in leaf color and conductance of mesophyll layer to CO_2_ during the drought stress period (Lauteri et al., 2014). The change in green color is due to the relative chlorophyll content in rice leaves and can be measured by SPAD meter under drought conditions in comparison to the irrigated conditions (Ndjiondjop et al., 2010; Barnaby et al., 2019). Zinolabedin et al. (2008) reported contradictory observations regarding water and nutrients uptake by the plant root system, which in turn caused a reduction in the chlorophyll concentration and subsequent yellowing of the leaves. Under drought stress, plants roll their leaves to reduce the transpiration rate, to maintain water levels (Sié et al., 2008). Leaf rolling capacity is now considered to be one of the major physiological adaptation strategies by crop plants to maintain their water status during drought stress periods (Fukai and Cooper, 2002). Moreover, a direct correlation was observed between grain yield and leaf rolling attributes of crop plants. Henderson et al. (1995) reported that leaf tip drying acts as a good indicator of the level of drought stress. The rolling of leaves is a reversible physiological adaptation, even though leaf tip rolling induced by drought stress is irreversible. Other abiotic stress factors, such as freezing and salinity, are also linked to a decline in the availability of water to plants. The decline in water availability is quantified as a decrease in the water potential. The indicator of dehydration avoidance is recognized by the leaf water potential (LWP) (Pantuwan et al., 2002). The stomata are closed when the water level goes beyond the water deficit in leaves to lower the rate of transpiration by minimizing the damage caused by water loss. Moreover, the higher LWP is regulated by varietal differences in the stomatal response and the closure of stomata (Jongdee et al., 1998). Higher photosynthetic capability possessed by certain genotypes helps plants to protect from the onset of senescence under stress conditions. This so-called stay green trait possessed by plants also helps in assimilating nitrogen and helps in optimizing the photosynthetic capability of plants under drought stress (Borrell et al., 2001). Other physiological adaptations possessed by plants include modifications in water uptake, loss of water potential, enhancing accumulation of compatible solutes, overexpression of tolerant proteins, prevention of cell damage and enhancing repairing by cell division.

### Root Traits

Imperative to the growth and metabolism of plants is the absorption of water and nutrients, which is grossly dependent on modifications in the morphological and physiological characteristics of roots (Ghosh and Xu, 2014). Various characteristics such as, type of root system, its properties, structures and wide distribution of roots helps to uptake water from soil to maintain productivity under water stress conditions (Comas et al., 2013). Under drought stress, several root traits such as, long specific root length; root length density and small fine root diameters maintain the productivity and regulate water levels in rice crops (Comas et al., 2013). For example, under drought stress periods, it was reported that rice tolerant varieties such as, Chuanguyou208 and Deyou4727 showed larger length in roots, higher root number and weight under 30–50 cm depth soil layer and in addition these varieties also displayed higher activities of peroxidase (POD) and superoxide dismutase (SOD) (Wang X. et al., 2019). Moreover, the hydraulic properties of roots in rice under drought stress were enhanced by developing deep and thick root systems (Lipiec et al., 2013). These attributes helped rice plants to develop tolerance to drought through absorption of water from deep layers of soil (Gowda et al., 2011; Uga et al., 2013a). Moreover, supplementation of rice seedlings with NH_4+_ resulted in higher root growth and an increased number of root tips to enhance water uptake under water stress conditions (Jia et al., 2020). In addition, rice seedlings supplemented with NO^3–^ induced restriction in water uptake resulted in the induction of root aerenchyma formation (Yang et al., 2012). Later studies revealed that NH4^+^ helped in higher water uptake compared with NO^3–^ due to enhanced root growth.

### Inflorescence Traits

Rice plant is susceptible to water stress during reproductive stage, and the severity of yield and quality losses depend on magnitude and duration of the stress (Yang et al., 2019; Chen M.-H. et al., 2021). Shaibu et al. (2015) compared continuous flood with two AWD schemes focused on two different flowering stages: AWD up to the start of flowering (AWD1), and AWD up to the start of grain filling (AWD2). The results showed that both AWD schemes had significant yield loss compared with the continuous flood treatment. The results emphasize the need for robust inflorescence traits for drought tolerance. Under drought conditions, it is interesting to note that generally early morning period is cooler compared to late morning and afternoons. Hirabayashi et al. (2015) identified a novel QTL, qEMF3, for early-morning flowering trait from wild rice, *Oryza officinalis*. The qEMF3 shifted flower opening time by 2 h earlier and improved spikelet fertility under stress environment. Similarly, studying pistil’s role is another interesting inflorescence trait that has not been explored in detail to unravel molecular basis of stress tolerance (Wang et al., 2021). Rice is one of the best models to study the development of inflorescence through molecular analysis of inflorescence mutants for investigating architecture of inflorescence (Zhang and Yuan, 2014). The critical factors to decipher the complexity of rice traits include the total number of fertile tillers, and the development and architecture of the inflorescence (Ikeda et al., 2004). The rachis, primary and secondary branches, including the spikelet, are critical physiological features of rice inflorescences. The spikelet consists of one fertile floret, two sterile florets and two rudimentary glumes. Spikelet differentiation, the determining factor of seeds per panicle, depends on the establishment, transition and activity of axillary and apical meristems. The development of rice inflorescences is well explained by the ABCDE model for the specification of floral organs in addition to the role of genes such as OsMADS3, OsMADS14, OsMADS15, and OsMADS58 (Yoshida and Nagato, 2011). The inflorescence in rice is controlled by cytokinin and auxin signaling pathways, CLV-WUS signaling and expression of MADS box genes. In rice, the Drought and Salt Tolerance (DST) protein, a member of zinc-finger transcription factor, induces overexpression of OsCKX2 in the reproductive meristem for induction of inflorescence (Li et al., 2013). The depth in understanding the molecular mechanism underlying the regulatory responses against drought and that influence the inflorescence development will further reveal deep comprehensions regarding enhancing yield and thus food security in the changing climatic scenario.

## Omics Approaches for Understanding Drought Tolerance

The methodologies studied under the omics approaches are categorized as genomics, transcriptomics, proteomics, metabolomics, epigenomics and comparative genomics.

### Genomic Approaches

Genomic studies are technical approaches for investigating the gene structure and functional dynamics of coding and non-coding sequences applicable to augmenting crop improvement. Important resources in structural and genomics studies include mutant libraries, cDNAs, expression profiles, sequence data sets and quantitative trait loci (QTLs) (Jiang et al., 2011). For instance, Vikram et al. (2012) cloned several genes and identified QTLs that were important for drought tolerance in rice. Large numbers of QTLs were identified to resolve the complex nature of drought stress and alleviating mechanism for tolerating drought stress (Fleury et al., 2010). In addition, several QTLs are identified to be responsible for enhanced grain yield and for other secondary characteristics that allow withstanding drought stress in rainfed uplands and lowlands (Bernier et al., 2007; Venuprasad et al., 2009). Differential performance of QTLs has been observed in upland and lowland ecosystems under drought conditions. Finally, the best suitable QTLs are selected as per the ecosystems, genetic background and environmental conditions. Moreover, for developing high yielding varieties, marker assisted back crossing (MABC) was efficaciously applied experimentally in crop plants. For example, MABC has been employed to increase the productivity of KDML105 rice variety in north-eastern regions of Thailand (Kanjoo et al., 2012). It is expected in near future that applications of molecular breeding techniques such as, marker-assisted selection (MAS), SNP marker applications and genome wide assisted selection (GWAS) will pave way to understanding the molecular mechanisms underlying the resilience in crop plants against wide range of environmental challenges such as drought stress.

### Comparative Genomics

The evolutionary history of organisms is clearly revealed by carrying out comparative account on structure and functions of genes across the species. For example, the phylogenetic relationships of Poaceae family (members of grasses) are now well understood by investigated species-specific DNA markers. Further, the comparative studies of genomes have led to identify the syntenic regions of different species. For instance, it is found that two thirds of genes located on chromosome number 11 in wheat are distributed in 06 homologues of grass genomes (Singh et al., 2004). The QTL1.1 is classical example of syntenic relationship; this QTL is found in chromosome 3 in maize, chromosome number 4B in wheat and chromosome 6 in barely (Swamy et al., 2011). The rice drought grain yield QTL was shown to be homologues of with maize counterparts (Swamy et al., 2011). Comparative analyses showed the role of OsCPK9 and AtCPK10 genes in augmenting the ABA dependent drought responsive mechanism (Zou et al., 2010; Wei et al., 2014). Whereas, OsCPK10 played pivotal role in defending cellular membranes against ROS and drought stress (Kumar et al., 2014; Nakabayashi et al., 2014; Fang et al., 2015; Yin et al., 2015). Large numbers of stress responsive genes are reported to play critical role in development and growth of plants. In addition, a network of genes pertaining to seed development and stress response has been reported in rice (Cooper et al., 2003). Molecular chaperons have been found to play critical role in circumventing the drought response in crop plants. Upon 2DE-MS and GC/MS based proteomic and metabolomic techniques, it was reported that Bip (molecular chaperon binding protein) was overexpressed in transgenic soybean plant (Coutinho et al., 2021). Moreover, transgenic rice over expressing *OsSKIPa* gene performed well under drought conditions due to its critical role in expression of series of stress related genes including, PP2C, CBF2, RD22 and SNAC1 and also enhancing the ROS scavenging ability in rice plants (Hou et al., 2009). Furthermore, *OsbZIP46CA1* transcription factor gene, a modified product of OsbZIP46 was found to be overexpressed in transgenic rice under drought stress, was found to enhance the expression of stress related genes, most of which in turn downregulate the ABF/AREBs (Ning et al., 2012). The transgenic approaches thus lead to unraveling the repository of genes critical for regulating plant metabolism under drought and other stress conditions, thus paving a way toward global food security (Details regarding genes conferring drought tolerance in rice have been presented in Table 1).

## Stress-Induced Regulatory Genes and Quantitative Trait Loci for Drought Tolerance

Against the stress induction, crop plants initiate a coordinated series of cellular and signaling processes. A great deal of physiological and morphological changes is actually the outcome of cellular responses regulated by large number of genes. It is reported that in rice, about 6,000 genes are downregulated and 5000 genes are upregulated under drought stress conditions (Maruyama et al., 2014). In addition, genome wide expression analysis led to identification of about 5,284 differentially expressed genes under drought conditions (Wang et al., 2011). For instance, the CO-like gene, *Ghd2* (grain number, plant height, and heading date2) is attributed to enhance the yield potential in rice. The *Ghd2* is almost prototypical to *Ghd7*, which helps in leaf senescence and drought tolerance. Contrary to this, it is reported that *Ghd2* overexpression considerably decreased the drought tolerance and once knocked-out it resulted in opposite effects (Liu et al., 2016). Another gene OsbZIP42 encoding a transcription factor is positive regulator of ABA dependent signaling pathway, hence critical for drought tolerance was found activated by stress or SAPK4 (ABA-activated protein kinase 4) (Joo et al., 2019). The OsbZIP42 genes, a member of EbZIP are found to be critical for established tolerance against the abiotic stress. In addition, large number of signaling cascades have been reported to be activated by ABA to activate adaptive response against abiotic stress conditions (Jiang and Zhang, 2002; Salazar et al., 2015; Sah et al., 2016).

In rice, SNAC1 and DST transcription factors (TFs) are activated by ABA mediated signaling to regulate stomatal movement (Hu et al., 2006; Huang et al., 2009). Furthermore, SNAC1 (stress responsive NAC1), a TF, is a member of the NAC family of TFs, which includes ATAF, CUC, and NAM, and approximately 149 members have been identified in rice plants. SNAC1 in particular is abundantly expressed in guard cells under drought stress conditions. Apart from ABA mediated activation of drought tolerant pathways, DREB (drought responsive element binding) is activated to act as frontline TFs to withstand drought stress (Yamaguchi-Shinozaki and Shinozaki, 2005). Moreover, the TF C_2_-H_2_-type zinc finger-containing protein helps in drought and salt tolerance and mediates H_2_O_2_ induced pathways to mediate stomatal closure (Huang et al., 2009).

The ABA phytohormone is activated by drought stress, which in turn causes stomatal closure and expression of diverse genes. The concentration of ABA in guard cells directly influences the closure of stomata, and its concentration is increased during drought stress. The pyrabactin tolerance (PYR)/PYL (PYR1-like)/regulatory receptors that belong to ABA response receptors sense ABA to initiate the downstream signaling cascade (Ma et al., 2009; Park et al., 2009). Upon ABA binding, its receptor undergoes conformational changes, which enable binding and inactivation of PP2Cs (protein phosphatase 2C), a negative regulator, including ABA insensitive 2 (ABI2), ABA insensitive 1 (ABI1), PP2CA and homology to ABI1 (HAB1) (Geiger et al., 2009). Since the negative regulator of the signaling pathway PP2C is inhibited, downstream signaling molecules are released their by switching the signaling cascade. The Ser/Thr kinase OST1 (open stomata1/SnRK2.6/SRK2E) is the main target of PP2C and is an important junction for the regulation of transcriptional responses, including ABA and CO_2_ responses. Upon release of OST1, it activates several TFs and phosphorylation of several plasma membrane proteins, which results in the closure of stomata through promoters such as AtRBOHF and SLAC1 (Fuji et al., 2009; Sirichandra et al., 2009; Klingler et al., 2010). In rice, protein complexes such as RCAR5/OsPYL, OsPP2C30, OREB1, and SAPK2 act as signaling units regulated by ABA (Kim et al., 2012). Among these proteins, OsPYL/RCAR5 were reported to be positive regulators of abiotic stress and are attributed to enhanced drought tolerance when overexpressed in transgenic rice plants. In rice, both reverse genetics and forward genetics have led to the identification of hundreds of stress responsive genes and respective QTLs (Wang et al., 2016).

A large number of kinases, such as OsMAPK5, which are functionally characterized as stress responsive MAPK (mitogen-activated protein kinase), have been found to be involved in regulating a diverse range of abiotic stresses, such as drought, cold and salt stress, even though they have been found to negatively regulate biotic stress, including bacterial and fungal infections (Xiong and Yang, 2003; Rohila and Yang, 2007). It has been reported that overexpression of TFs such as, SKIP, bZIP, and NAC helps to enhance drought tolerance in rice. In particular, NAC TFs, such as OsNAC5, OsNAC6, OsNAC9, OsNAC10, OsbZIP16, OsbZIP23, OsbZIP46, and OsbZIP71 are involved in enhancing drought tolerance in crop plants (Fukao and Xiong, 2013). In addition, WRKY TFs are also involved in drought tolerance and a large number of growth and developmental processes. For example, 97 WRKY genes identified in *O. nivara* (OnWRKY) and 89 WRKY genes in *Japonica* were mapped and identified in plants that have diverse range of functions (Sahebi et al., 2018).

A large number of QTLs have been identified in different populations linked to drought tolerance (Khowaja and Price, 2008). Large effect QTLs have also been identified for grain yield under drought conditions (Bernier et al., 2007). Approximately, 77 QTLs for grain yield and other aspects important for drought tolerance were identified by crossing two rice cultivars (Lanceras et al., 2004). These QTLs were identified to possess several physiological attributes, such as 05 QTLs for days to flowering, 6 QTLs for harvest index, 7 QTLs for grain yield, 7 QTLs for percent spikelet sterility, 8 QTLs for biological yield, 10 QTLs for total spikelet number, 11 QTLs for plant height and 23 QTLs for panicle number. Approximately 10 QTL components and high grain yield were identified by using a recombinant inbred population of rice, such as IR64 and Cabacu, under drought stress (Trijatmiko et al., 2014). The QTL, *qDTY12.1* was the first report linked to grain yield in the upland reproductive stage under drought conditions. This QTL was identified from the 436 F3 populations derived from Vandana and Way Raren (Bernier et al., 2007). The *qDTY2.1* and *qDTY3.1* identified in the backcross between Swarna and Apo are two other large effect QTLs known to affect grain yield under drought conditions at the lowland reproductive stage. Both QTLs showed pleiotropic effects on a few traits, such as PHT (plant height) and DTF (days to flowering) (Venuprasad et al., 2009). Moreover, Venuprasad et al. (2012a) reported *qDTY6.1* QTL has had a strong effect on aerobic drought stress. Grain yield under severe drought stress in lowland reproductive stage QTLs was identified in F3 populations derived from the crosses of Swarna × N22, IR64 × N22 and MTU1010 × N22 (Vikram et al., 2011). Later, QTLs were also identified in the Apo/IR64 and CT9993-5-10-1-M/IR62266-42-6-2 populations (Kumar et al., 2008; Venuprasad et al., 2012b). Other QTLs reported include *qDTY2.2, qDTY4.1, qDTY9.1*, and *qDTY10.1*, which were linked to grain yield and identified in populations obtained from backcrosses between IR64 and Aday Sel rice varieties (Swamy et al., 2013) (Details related to QTLs conferring drought tolerance in rice have been presented in Table 2).

## Root Architecture for Drought Tolerance

Water deficit is overcome by a series of genes responsible for root architecture and hence higher yields are observed in crop plants (Jeong et al., 2010; Iwata et al., 2013; Cheng et al., 2015). Specifically, the NAC family of TFs was found to be overexpressed, to regulate root architectures (Zheng et al., 2009). For instance, OsNAC9 is a member of the NAC TF that is responsible for root architecture under drought tolerance and grain yield (Redillas et al., 2012). Later, this gene was reported to be overexpressed when controlled by a constitutive or root specific promoter in transgenic rice under both drought and well-watered conditions. It was reported that recombinant lines of maize have 144% more yield than control plants under drought conditions (Zhan et al., 2015). In rice, the overexpression of the *OsDHODH1* gene, encoding a putative cytosolic dihydroorotate dehydrogenase (DHODH), enhanced drought and salt tolerance (Liu et al., 2009).

Enormous putative genes controlling physio-morphological traits confer drought tolerance in rice (Deivanai et al., 2010). The appropriate water and nutrient status of plants is largely supported by the root architecture (Kato et al., 2006). The rooting depth, root thickness, root density and distribution pattern of roots are various traits studied under root system architecture to regulate water and nutrient uptake to address adverse conditions of drought (Lilley and Fukai, 1994; Fukai and Cooper, 1995; Pantuwan et al., 1996; Wade et al., 1996). Furthermore, in rice populations, various QTLs related to morphology and root index penetration have been identified (Champoux et al., 1995; Ray et al., 1996; Zhang et al., 2001; Henry et al., 2014; Kijoji et al., 2014). Important traits adapted by crop pants to retain water and nutrients during drought stress include the ratio of deep rooting (RDR), root growth angle (RGA) and direction of root elongation. In rice plants, overexpression of *EDT1/HDG11* genes resulted in enhanced root development and reduced stomatal density under drought conditions (Yu et al., 2013). For instance, squalene synthase (SQS), a key enzyme located in the endoplasmic reticulum is capable of catalyzing first reaction, i.e., conversion of two farnesyl pyrophosphates into squalene, a step in the isoprenoid metabolic pathway finally directed to synthesize sterols in plants (Tansley and Shechter, 2001). Under drought conditions, it was reported that the disruption of squalene synthase (SQS) function by RNAi led to enhanced root length, a higher number of lateral roots and a decline in the stomatal conductance (Manavalan et al., 2012).

Approximately 675 QTLs for root traits were identified by meta-analysis of 12 populations of crop plants (Courtois et al., 2009). For deep rooting traits, only 05 major QTLs were mapped as root traits (Kitomi et al., 2015; Uga et al., 2011, 2015). A cross between the deep rooting cultivar Kinandang Patong and the shallow-rooting cultivar IR64 resulted in recombinant inbred lines (IK-RILs) leading to the identification of the DRO1 gene located on chromosome 9 (Uga et al., 2011). It was reported that DRO1 considerably encodes the RGA and grain yield. The DRO2 gene found on chromosome 4 is another major QTL that encodes the RGA and was obtained by crossing Kinandang Patong and shallow-rooting cultivars (ARC5955, Pinulupot1 and Tupa729) (Uga et al., 2013b). Furthermore, the DRO3 gene found on chromosome 7 also had a great impact on the RGA due to its involvement in the DRO1 genetic pathway (Uga et al., 2015). Genome wide expression profiling also led to the identification of *OsAHL1*, a novel gene involved in drought tolerance and avoidance in rice plants. Later, genes were found to regulate root development, oxidative stress and chlorophyll content under drought stress conditions (Zhou et al., 2016). Norton and Price (2009) identified 04 QTLs for seminal root morphology and 2 QTLs for root gravitropic responses. By using 124 recombinant inbred lines, QTLs such as Soil Surface Rooting 1 (qSOR1) were found on chromosome 7 in rice (Uga et al., 2012). In addition, 2 QTLs for canopy temperature and 6 for leaf water potential in RILs were identified in crop plants (Liu et al., 2005). Lou et al. (2015) identified 6 QTLs by using 1,019,883 SNPs. In drought stress, 1 QTL for leaf drying, 1 for SPAD and 2 QTLs for canopy temperature were identified to manage stress (Prince et al., 2015). It is concluded that the introduction of traits contributes to drought tolerance and avoidance in rice to enhance yield (Fukai and Cooper, 1995; Nguyen et al., 1997). Moreover, a considerable number of QTLs have been mapped for osmotic adjustment in crop plants, even though very few loci have a major impact during stress conditions (Lilley et al., 1996). On the other hand, DRO1 was found to enhance yield under drought stress (Uga et al., 2013a).

Genomics plays a pivotal role in deciphering the new genome sites that code for critical traits and apprehending the nucleotide variations linked to specific variant phenotypes. Specific Rice SNP-seek database was updated to gain insights into nucleotide and phenotypic variants (Locedie et al., 2017). The nucleotide variants were curated by databases of rice such as, Gramene (Tello-Ruiz et al., 2016), NCBI (Sherry et al., 2001), RiceVarMap (Zhao et al., 2015), RMBreeding (Zheng et al., 2015), and IC4R (The IC4R Consortium, 2015).

## Transcriptomics

Transcriptomic studies involve studying total transcripts found in cells/tissues or organisms. Through transcriptome-wide studies, it has been revealed that a large number of TFs have helped to confer drought tolerance in rice. Broadly, two classes of TFs have been identified to mediate signaling and physiological responses in plants such as, ABA independent and ABA dependant activation of TFs for drought tolerance. ABA dependant TFs includes (1) basic leucine zipper (bZIP) and (2) NAM, ATAF, and CUC2 (NAC). The bZIP class of TFs plays a pivotal role in dehydration induced by ABA signaling under drought conditions (Yang et al., 2010). Moreover, ABF-3 mediated drought tolerance was more efficient than *DREB1A/CBF3* triggered drought stress tolerance (Oh et al., 2005). NAC TFs are another potential member that belongs to the ABA dependant group, which consists of a DNA binding NAC domain (Palaniswamy et al., 2006; Guo et al., 2008). The SNAC1 is typical of the NAC family and is overexpressed in transgenic rice lines showing a higher seed setting rate and spikelet fertility under severe drought stress (Hu et al., 2006).

Two families of TFs are discussed in ABA-independent signaling pathways, zinc fingers and AP2/ERFs. Former TFs consist of Zn ions coordinated to motifs that stabilize the protein folds. These TFs include *WRKY, ZFP252*, and *Zat10/STZ* genes (Xu et al., 2008; Wu et al., 2009; Xiao et al., 2009). Among these, STZ was reported to enhance the fertility of spikelets and grain yield under drought stress (Xiao et al., 2009). Under the control of the drought inducible promoter *OsHVA22P*, *AtDREB1A*/*CBF3* gene overexpression enhanced drought tolerance in rice plants to increase the yield and spikelet fertility. Large numbers of TFs are deployed to impart drought tolerance in crop plants, but their successful introduction in crop plants is still in its infancy.

Tian et al. (2015) assembled the *de-novo* transcriptome of common wild rice (*Oryza rufipogon* Griff.) and identified several drought responsive and related genes in root tissue based on transcriptomic data, thus, providing critical data pertaining to genetic and genomic studies in rice. Analyzing the transcript and metabolic responses of two rice cultivars with contrasting drought-tolerance to long-term drought, Ma et al. (2016) showed that a large number of DEGs related to photosynthesis were upregulated in the drought tolerant variety and concluded that well-maintained photosynthesis under drought is a critical aspect for improved drought-tolerance in rice. An RNA-seq based transcriptomic profile between OsMIOX-overexpressing (OE) and wild-type (WT) rice plants revealed that this unique monooxygenase plays an essential role in drought tolerance. Significantly, upregulated DEGs in OE lines were associated with TFs, plant hormone transduction and sugar metabolism (Shi et al., 2020). Luo et al. (2020) examined the morphological differences between upland and lowland rice ecotypes. Furthermore, genetic and transcriptomic divergences between these contrasting rice ecotypes under well-watered and drought conditions were observed using RNA-seq. The results revealed that the expression divergences were higher in upland rice than in lowland rice, and it was concluded that this transcriptomic divergence contributed to their morphological differences in drought tolerance.

Xia et al. (2020) investigated the temporal transcriptomic data of 12 rice genotypes varying in naturally induced drought in field conditions. They highlighted that the drought tolerant varieties had higher proportions of upregulated DEGs related to fucose, trehalose, and raffinose metabolic processes that were specifically induced. In addition, the DEGs related to proteins and their modifications such as protein peptidyl-prolyl isomerization, histone deacetylation, transcriptional attenuation and ferric iron transport, were induced earlier in drought tolerant genotypes. An integrated phenotypic and transcriptomic landscape of 61 rice (*Oryza sativa*) varieties grown in an upland field with highly diverse below-ground traits under mild drought stress was reported (Kawakatsu et al., 2020). Rice accessions were classified into four admixture groups based on phenotypic variation and the transcriptomic analysis showed admixture group-specific enrichment of stress-related genes. Signaling network and key TFs were identified by co-expression network analysis of DNA affinity purification followed by sequencing (DAP-seq) datasets and were found to negatively regulate crown root diameter. Tarun et al. (2020) carried out co-expression network analysis of the transcriptomes obtained from emerging panicle tissues and flag-leaf in drought-tolerant yield recurrent parent and introgression line under drought stress. Results indicated that plants scavenge ROS and enhanced protein turnover as pivotal mechanisms induced in both tissues during drought stress conditions.

Liang et al. (2021) conducted comparative RNA-seq analysis of a moderately tolerant breeding line and drought susceptible elite varieties during the grain-filling stage and observed that the tolerant line had much earlier responses at the transcriptomic level. Genes and gene families related to TFs, drought tolerance genes, and reactive oxygen species (ROS) scavengers were significantly altered. More recently, Tiwari et al. (2021) carried out time-based comparative transcriptomic profiling of two varieties that showed contrasting drought tolerance. Comparatively, a significant proportion of the identified DEGs related to phytohormone signaling, stress-response, photosynthesis, anti-oxidative mechanisms and TFs were higher in the drought tolerant variety. Furthermore, QTL mapping could distinguish drought-responsive traits at the chromosomal level, because a very high number of DEGs associated with drought stress was observed in the drought tolerant variety.

Studies have revealed that to unravel the complexity of expression levels, it is important to investigate the transcriptomes as a function of development and environmental conditions (Dayan et al., 2015; Liu et al., 2015; Gould et al., 2018). Furthermore, the transcriptomic investigation of rice lines under two different drought stress conditions could help to elucidate the possible molecular switches adapted by rice crops (Gould et al., 2018). The variation induced by stress tolerant and stress sensitive cultivars of rice at the molecular level was identified by microarray analysis (Walia et al., 2005; Lenka et al., 2011; Ray et al., 2011). The studies evaluated the differential expression of genes by both tolerant and sensitive cultivars of rice under stress conditions (Walia et al., 2005; Lenka et al., 2011). Therefore, it is important to gain in-depth knowledge about the complexity of transcriptional regulation in rice during drought and salinity stresses using advanced technologies.

## Proteomics

This approach involves studying the proteomes of a cell/organ/tissue and organism for experimental purposes (Rakwal and Agrawal, 2003). Proteomes are largely influenced by the environmental conditions that occur in the immediate vicinity of biological systems. Therefore, it is pertinent that genomes can produce a large number of proteomes according to the prevailing conditions. Little progress has been made in rice proteomics compared to genomic studies. Proteome maps of rice seeds, roots and leaves investigated at different developmental stages augment the development of stress tolerant rice varieties (Komatsu et al., 1999; Koller et al., 2002; Tanaka et al., 2005; Nozu et al., 2006). Approximately 31 drought responsive proteins have been identified in crop plants (Muthurajan et al., 2010). The preliminary Rice Proteome Database was available (Komatsu et al., 2004; National Institute of Agrobiological Sciences) and the data might be used to process the comparative proteomic analysis of drought tolerant and control varieties of rice.

A tandem mass tag (TMT)-based proteomic approach was applied to compare the roots, flag leaves and spikelets of a wild type and its near-isogenic line (NIL) harboring QTL qDTY 12.1, a large effect QTL for rice yield under drought (Raorane et al., 2015). The proteomic data correlated with drought-specific morpho-physiological responses and tissue-specific differences in protein abundance were observed. The DAPs were related to respiration, photosynthesis, energy generation and carbon-nitrogen acquisition/remobilization largely through the pathways of sugar/starch and amino acid metabolism. Paul et al. (2015) carried out comparative proteomic analysis to understand the metabolic networks regulated by the proteomes of roots in transgenic and wild type rice plants. Later studies led to the identification of DREB1A overexpression in rice plants by employing two-dimensional gel electrophoresis (2DE)-coupled with MALDI TOF MS/MS cultivated under drought stress. Most DAPs were linked to carbohydrate metabolism and defense, and a novel protein, R40C1, significantly accumulated only in the roots of transgenic plants. Agrawal et al. (2016) employed the 2DE-MALDI-TOF-MS/MS approach to compare the root cytoplasmic proteome of a drought tolerant rice variety for drought tolerant rice grown under normal and PEG-induced drought conditions. Major proportion of the identified differentially abundant proteins (DAPs) were involved in bioenergy, metabolism, cell defense and rescue, protein biogenesis, protein storage and cell signaling, and the authors proposed that protein biogenesis, cell defense, and efficient homeostasis could render better drought-adaptation. Wu et al. (2016) carried out comparative label-free shotgun proteomics and TMT labeling proteomics of two rice cultivars with contrasting genetic backgrounds and levels of tolerance to drought. Proteomics results indicated alteration in multiple stress and defense response related proteins. Notably, a ClpD1 protease was upregulated several folds only in the drought tolerant variety, and porphyrin and chlorophyll biosynthesis pathways were down-regulated. A comparative proteomic analysis of a susceptible rice cultivar and its stress-resistant somaclonal mutant line showed that a large proportion of DAPs primarily related to retrotransposons were predominantly identified in the resistant line, suggesting that the gene expression associated with drought tolerance mechanisms is under strong epigenetic regulation. It was also suggested based on proteomic analysis that photosynthetic adaptation through NADP(H) homeostasis contributes to drought tolerance in rice (Chintakovid et al., 2017).

Wang et al. (2017) performed physiological measurements and conducted 2DE-based proteomic analysis of rice flag leaves at the flowering and milk stages to understand the drought responsive mechanism. Based on the proteomic analysis, it was postulated that at the flowering stage, CO_2_ assimilation and ATP synthesis were disrupted, while at the milk stage, both CO_2_ assimilation and photosynthesis were impaired. However, there was an increased abundance of DAPs related to defense and antioxidant machinery, which suggests redox imbalance and activation of the ROS scavenging system in drought stressed plants (Saini et al., 2021).

To understand the role of jasmonic acid under drought stress, studies were conducted to compare the morpho-physiological traits and the root proteome of a wild type (WT) rice plant with its jasmonic acid biosynthesis mutant *coleoptile photomorphogenesis 2* (*cpm2)*, disrupted in the allene oxide cyclase (AOC) gene. Studies revealed that roots of *cpm2* mutants compared to wild type had higher water use efficacy, high ABA concentration in shoots, and higher stomatal conductance under drought conditions. Moreover, the TMT technique was used to analyze the root proteome to better understand this difference at the molecular level. Identification of other DAPs proposed increased energy metabolism (i.e., increased mobilization of resources) and ROS scavenging in *cpm2* under drought. In addition, it was revealed that there was an abundance of proteins pertaining to cell growth, secondary metabolism and cell wall synthesis in *cpm2* mutants grown under drought stress. A clear understanding of adaptation and responses in roots of *cpm2* mutants under drought stress was elucidated by proteome-guided metabolites, transcripts and histological investigations (Dhakarey et al., 2017). By conducting comparative morphological and proteomic analysis of two rice genotypes, 5 DAPs, including chitinase, were found to be abundant under drought stress (Anupama et al., 2019).

Cunha et al. (2019) investigated APX found in rice thylakoids in response to mild drought stress by carrying out physiological measurements and leaf proteomic analysis of thylakoidal APX knockdown rice plants (apx8) and non-transformed control plants. A correlation was observed between the sensitivity of plants to mild drought stress and the lower accumulation of DAPs related to several metabolic processes, especially photosynthesis, photorespiration and redox metabolism, in *apx8* plants. Although *apx8* effectively induces other compensatory antioxidant mechanisms in well-watered conditions, the *apx8* plants could not maintain H_2_O_2_ homeostasis and accordingly avoid adverse conditions under mild drought conditions. To carry label-free proteomic studies, Hamzelou et al. (2020) exposed eight genotypes of upland and lowland rice plants to drought stress at the late vegetative stage. The majority of the identified DAPs under drought conditions were related to photosynthesis, oxidative stress response, proteolysis, and translation of stress-responsive proteins, such as heat shock, and LEA proteins (late embryogenesis-associated proteins) sharply increased under drought stress. Du et al. (2020) investigated the physiology of rice grown under two nitrogen management modes by proteomics and metabolomics approaches to investigate their yield formation and the mechanism of nitrogen regulation for drought tolerance. It was evident from the proteomic analysis that the most altered biological processes and pathways were related to the biosynthesis of primary and secondary metabolites important for the growth and metabolism of crop plants.

The rice proteomes are assessed by several gel and off-gel based proteomics techniques. For instance, proteome changes are analyzed by a combination of proteomic techniques such as 2-DE matrix-assisted laser desorption ionization-tandem time of flight (MALDI-TOF/TOF) and 1-DE/LC-Fourier transform-ion cyclotron resonance (FT-ICR) MS based analyses (Wan and Liu, 2008). Moreover, several proteomic databases for rice have been established and updated to curate the sequences for desirable traits. For example, the Rice Yield-related Database (RicyerDB) was created to curate the research related to yield related traits in rice by assessing the genomic and proteomic data inputs, and it provides a vast literature source related to where we can browse, investigate and analyze the desirable genes related to yield (Jiang et al., 2018). Systemic proteome analysis through a wide range of proteomic techniques is a powerful tool to investigate the complete proteomes of rice plants at different levels, such as the organelle, cell, tissue, organ and organism levels, and further provides insights into assessing proteomes once plants are exposed to stressful conditions.

## Metabolomics of Rice Plants

Metabolomic studies involve comprehensive and systematic identification and quantification of endogenous metabolites from biological samples and have rapidly evolved and advanced in recent years (Ryan and Robards, 2006; Kumar et al., 2017).

### Some Major Breakthroughs in Metabolomics-Related Articles on Rice From 2015 to 2021

These include: Nam et al. (2016) – studies carried in transgenic rice to unravel the metabolomic changes in grains of well-watered and drought conditions*;* Ma et al. (2016) – studied the key pathways by transcriptomic and metabolomic studies to maintain photosynthesis under the drought and the consequent drought-tolerance in rice*;* Xiong et al. (2019) – Analysis of grain yield reduction in rice by comprehensive proteomic, metabolomic, and physiological analyses of grain under abrupt drought–flood alternation stress*;* and, Du et al. (2020) – Combinatorial proteomic, metabolomic and physiological studies of rice growth and grain yield with heavy nitrogen application before and after drought.

Metabolomics analysis in plant systems is rapidly advancing because it measures total, or groups of metabolites expressed in few samples in specific time-periods. Metabolomes of higher plants could comprise hundreds of thousands of metabolites out of that number, so little is known (Wang S. et al., 2019). Qualitative and quantitative measures of plant metabolomes mirror their responses to organic phenomenon and abiotic stimuli, genome, and physiological standing, serving as a connecting link between genotypes and phenotypes. They provide a considerable contribution to stress biology by deciphering numerous compounds such as by-products of stress metabolism, signaling molecules, and compounds, that square measure a part of the plant acclimatization method (Lanzinger et al., 2015; Ramalingam et al., 2015). Metabolites such as amino acids, fatty acids, soluble sugars, nucleotides, organic acids, phenolics, peptides, cofactors, and secondary metabolites act as cellular measurements that changed during drought stress (Das et al., 2017; Arora et al., 2018). Various metabolomic approaches elucidated the wide range of metabolite markers expressed in response to abiotic stressors (Ghatak et al., 2018). Several of these metabolites square measure crucial parts of the plant’s weapons system. Polyamines and phenoplast compounds are major substantial plant secondary metabolites that impart tolerance, and square measures are interpreted as replacements for biostimulants underneath environmental stress, especially drought stress conditions (Aninbon et al., 2016; Chen D. et al., 2019). Underneath environmental stresses, metabolic changes could assert some necessary metabolic products, which illustrates that the metabolic pathways are regulated at different levels. Additionally, plants can also change metabolic pathways to accumulate giant amounts of energy, which is a prerequisite to resist environments (Ullah et al., 2017). Metabolic analyses have shown that changes in metabolites under drought responses in various plant species are pivotal for developing adaptations. In Arabidopsis, most amino acid intermediates (such as from proline, glutamine, tryptophan, alanine, aspartate, ornithine, isoleucine, leucine, and valine) from the TCA cycle (such as 2-oxoglutarate, cis-aconitate, and succinate), flavonoids (such as quercetin and cyanidin) and lipids accumulate under drought stress (Tarazon et al., 2015; Pires et al., 2016). Similarly, in rice the metabolomic data suggest that in response to increasing water stress, rice cultivars that were drought tolerant accumulated higher levels of carbohydrates (fructose, glucose, and myo-inositol) compared with the susceptible cultivars (Barnaby et al., 2019).

Both quantitative and qualitative rice metabolomics analyses help to investigate primary and secondary metabolites and their differential expression patterns during biotic and abiotic stress conditions (Khakimov et al., 2014). Major metabolic techniques, such as nuclear magnetic resonance (NMR), GC/MS, and LC/MS in combination with Fourier transform ion cyclotron resonance (FT-ICR), have been employed to decipher the metabolite profiles of organisms in conjunction with databases, including Kyoto Encyclopedia of Genes and Genomes (KEGG) and MetaCyc (Kanehisa et al., 2012; Morreel et al., 2014; Caspi et al., 2018; Chen L. et al., 2021). The database specific to rice, including RiceCyc, is constricted based on metabolic networking and MetaCyc (Chae et al., 2007). To comprehend the basic mechanism of the response to abiotic stress, in particular drought, comparative metabolomics approaches provide a solid framework of gene expression, protein expression and other metabolites expressed temporally. Metabolomics approaches are promising technical interventions that overall serve as frameworks to obtain an in-depth biochemical and genetic portrait of organisms to pave the way for molecular breeding under stress conditions (Bino et al., 2004; Fernie and Schauer, 2009; Kusano et al., 2011).

## Epigenomics for Drought Tolerance

The epigenome could be outlined as the summation of all of the biochemical changes in nuclear polymer, simple proteins and tiny non-coding ribonucleic acid biogenesis of a cell. Studies on the epigenetic changes in and around polymers that regulate ordination activity are outlined as epigenetics, and the branch of genetic science that addresses epigenomic studies is named epigenomics. Plants have evolved non-heritable, extremely subtle systems to deal with varied environmental stresses. The last decade has witnessed considerable progress in understanding the signaling and metabolic pathways that are dominant in plant responses to stresses, which has been summarized in previous reviews (Ku et al., 2018; Kumar et al., 2019). Activation of signaling pathways typically results in transcriptional adjustments to initiate the expression of stress responsive proteins (Kim et al., 2019; Shahid, 2019; Wu et al., 2019). A large number of adaptations have been attributed to crop plants in response to stress factors (Thomashow, 1999). The temporal responses to stress include the expression of stress-induced proteins, RNA molecules and metabolites. These stress adaptions could be longer if phenological and morphological adjustments have been accomplished. The transcriptional reprogramming and regulation of stress-responsive genes are critical to numerous epigenetic processes and components, such as DNA methylation, protein modifications and non-coding RNAs based regulations (Deleris et al., 2016; Kim et al., 2017; Chen R. et al., 2019). Drought stress conditions primarily tend to extend demethylation. It is conjointly discovered that DNA methylation shows tissue specificity. A variation of up to 1% methylation occurs across cells, tissues, genotype and organic process stages. The DNA methylation level is reported to be lower in roots than in leaves, which indicates an important role of roots in addressing water scarcity (Suji and John, 2010). The correlation between drought stress and DNA methylation is shown in lowland and drought-tolerant rice cultivars. The drought inclined IR20 variety showed hypo-methylation under drought conditions, whereas the tolerant varieties “Paiyur” and “PMK3” showed hyper-methylation. These changes in methylation patterns were found to be responsible for the differential expression of stress responsive genes (Gayacharan, 2019). In another study conducted in rice, it was illustrated that hypomethylation has an important role within the drought tolerance attributes of rice genotypes. Epigenomic studies involve a range of techniques, such as chromatin immunoprecipitation (ChiP), ChiP-sequencing, methylated-DNA immunoprecipitation and shotgun bisulfite sequencing. For the development of resilient rice cultivars, it is pertinent to focus on the profile and functioning of epigenomic profiles, such as histone modifications, DNA methylation, diverse classes of regulatory non-coding RNAs and especially the 3D genomic structure of rice (Lu et al., 2020). The role of epigenomics studies has considerably contributed to studying the changing dynamics of expression patterns under stress conditions, thus unraveling the complexity of crop plants in response to a wide range of stress factors. In addition, a detailed account on rice epigenomics variations and their identification is critical to the characterization of phenotypes fitting to agronomic traits for providing framework for enhancing productivity and traits in rice crops (Chen and Zhou, 2013).

## Conclusion

**FIGURE 4:**
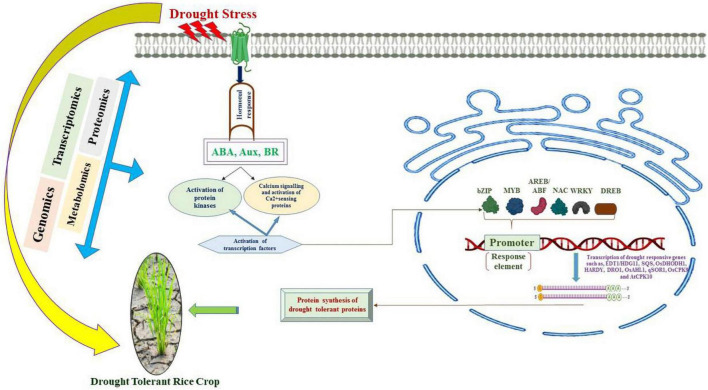
Signaling cascades activated due to drought stress that are involved in the expression of drought tolerance induced genes.

The mechanism of drought tolerance is an important quantitative trait that is accompanied by several phenotypic adjustments and adaptations. A large number of stressors are supposed to be tolerated by crop plants, such as high temperatures, high irradiance, toxicities, and nutrient deficiencies due to the induction of drought stress. The past few decades have witnessed a higher prevalence of stresses due to abiotic factors imparting hindrances to growth and productivity in rice crop. Globally, scientists are extensively investigating strategies to cope with ever increasing climatic change and drought stress factors. The emergence of innovation in omics technologies has led to the deciphering of a large amount of molecular machinery to adapt to a large number of environmental factors. Besides, multiomics data integration and molecular modeling protocols can be employed to understand the complex traits to unearth the tolerance mechanism and yield of major legumes and cereal crops (Pazhamala et al., 2021; Yang et al., 2021). It is pertinent that enhancing tolerance to drought in rice requires the establishment of research programs to investigate complex networks of molecular interactions. Investigations based on transcriptomics, proteomics and metabolic studies should be initiated in unison to unravel the drought tolerance mechanisms in rice. These techniques will further widen our understanding of molecular signaling cascades and a wide range of stress responsive proteins and intracellular adjustments under stress factors (Figure 4). Moreover, the cited literature validates the role of investigating QTLs and SNPs that play pivotal role of genes in drought tolerance in rice crops. This investigation at large could possibly augment our efforts to develop drought tolerant crop plants such as rice to ensure food security. The applications of markers and genomic selection are an efficient way to improve crop plants. Moreover, the development of high throughput phenomics techniques will help to augment the in-depth understanding of the mechanisms of tolerance to stressors. A low level of progress has been made with respect to proteomics, epigenomics and metabolomics in conjunction with genomic approaches. It is an important prerequisite to use the data obtained from these approaches to understand the genetic and physiological basis of drought tolerance. In addition, efforts aimed at combinatorial omics approaches and practical plant-breeding applications can accelerate progress in producing rice cultivars that are suitable for rainfed environments or to realize significant freshwater savings. Against this background, a PANOMICS platform that integrates mathematical and statistical tool boxes with omics data to facilitate improvement of crops through discovering pathways and target genes critical for resilience and creation of elite lines (Weckwerth et al., 2020). Finally, physiological and morphological adaptations accomplished by molecular mechanisms traced in resistant parent lines can be utilized to transfer the resilient genes to mainstream crop plants to become very adept to a particular environment, to establish a solid base for food security at a global level.

## Author Contributions

SZ and RR conceived the idea, framed outline and contributed in writing and final editing of the manuscript. RAM contributed in writing genomics part and prepared the figures. LE prepared transcriptomics part of the manuscript. AM edited the manuscript and also contributed in preparing the proteomics and transcriptomics sections. AH, MM, and RM prepared the tables and also contributed in preparing genomics section. RS and NS contributed in writing introduction and physiological section of the manuscript. JR revised the original draft and carried out the final editing with SZ and RR. All authors contributed to the article and approved the submitted version.

## Conflict of Interest

The authors declare that the research was conducted in the absence of any commercial or financial relationships that could be construed as a potential conflict of interest. The handling editor declared a past co-authorship with one of the authors RR.

## Publisher’s Note

All claims expressed in this article are solely those of the authors and do not necessarily represent those of their affiliated organizations, or those of the publisher, the editors and the reviewers. Any product that may be evaluated in this article, or claim that may be made by its manufacturer, is not guaranteed or endorsed by the publisher.

## Footnotes

1
http://www.knowledgebank.irri.org/training/fact-sheets/water-management/saving-water-alternate-wetting-drying-awd
